# Promoting traditional foods for human and environmental health: lessons from agroecology and Indigenous communities in Ecuador

**DOI:** 10.1186/s40795-020-00395-y

**Published:** 2021-01-07

**Authors:** Ana Deaconu, Ekomer, Stephen Sherwood, Myriam Paredes, Peter Berti, Pablo López, Donald Cole, Fabian Muñoz, Pedro Oyarzún, Ross Borja, Marcelo Aizaga, Eliana Estrella, Gabriel April-Lalonde, Geneviève Mercille, Malek Batal

**Affiliations:** 1 Transnut WHO Collaborating Centre on Nutrition Changes and Development at Université de Montréal, Faculty of Medicine, Nutrition Department, 2900, boul. Édouard-Montpetit, Montréal, Québec H3T 1J4 Canada; 2 Centre de Recherche en Santé Publique (CReSP), 7101, avenue du Parc, Montréal, Québec H3N, 1X9 Canada

**Keywords:** Traditional foods, Agroecology, Nutrition transition, Indigenous, Farmers, Diet, Andes, Ecuador, Production diversity

## Abstract

**Background:**

The displacement of traditional dietary practices is associated with negative nutritional consequences for rural Indigenous people, who already face the brunt of both nutritional inadequacies and excesses. Traditional food (TF) consumption and production practices can improve nutritional security by mitigating disruptive dietary transitions, providing nutrients and improving agricultural resilience. Meanwhile, traditional agricultural practices regenerate biodiversity to support healthy ecosystems. In Ecuador, Indigenous people have inserted TF agricultural and dietary practices as central elements of the country’s agroecological farming movement. This study assesses factors that may promote TF practices in rural populations and explores the role of agroecology in strengthening such factors.

**Methods:**

Mixed methods include a cross-sectional comparative survey of dietary, food acquisition, production and socioeconomic characteristics of agroecological farmers (*n* = 61) and neighboring reference farmers (*n* = 30) in Ecuador’s Imbabura province. Instruments include 24-h dietary recall and a food frequency questionnaire of indicator traditional foods. We triangulate results using eight focus group discussions with farmers’ associations.

**Results:**

Compared to their neighbors, agroecological farmers produce and consume more TFs, and particularly underutilized TFs. Farm production diversity, reliance on non-market foods and agroecology participation act on a pathway in which TF production diversity predicts higher TF consumption diversity and ultimately TF consumption frequency. Age, income, market distance and education are not consistently associated with TF practices. Focus group discussions corroborate survey results and also identify affective (e.g. emotional) and commercial relationships in agroecological spaces as likely drivers of stronger TF practices.

**Conclusions:**

Traditional food practices in the Ecuadorian highlands are not relics of old, poor and isolated populations but rather an established part of life for diverse rural people. However, many TFs are underutilized. Sustainable agriculture initiatives may improve TF practices by integrating TFs into production diversity increases and into consumption of own production. Agroecology may be particularly effective because it is a self-expanding global movement that not only promotes the agricultural practices that are associated with TF production, but also appears to intensify affective sentiments toward TFs and inserts TFs in commercial spaces. Understanding how to promote TFs is necessary in order to scale up their potential to strengthen nutritional health.

## Background

Globally, populations are hastily replacing their traditional food^1^ practices with diets marked by excesses in sugar, sodium, fat, and calories, and this pattern is accelerating among the world’s rural poor [2]. In the face of this nutrition transition [3], Indigenous people in Ecuador aspire to preserve their traditional food practices, which they perceive as being healthier, more resilient and more culturally meaningful than non-traditional foods [4]. However, biodiversity loss, dietary transitions and shifting agricultural strategies threaten their access to these products [4]. In localities around the world, traditional practices around food have been observed to be associated with balanced diets and dietary health [5–8], cultural integrity [5, 9], and resilient agricultural ecosystems, especially in the face of climate change [10, 11]. Such practices include the production of traditional crops and crop varieties; traditional agricultural techniques, including intercropping and high agricultural biodiversity; hunting, fishing and wild harvest of traditional foods; and, consumption of traditional foods on their own or as parts of dietary patterns [4–7, 9–11]. Yet the homogenizing march of globalization has made it be that traditional foods have in many cases become synonymous with “neglected” and “underutilized” crops, the former referring to crops ignored by the scientific community, and the latter referring to those that have largely fallen out of cultural and economic use [7, 12].

The decline of traditional food (TF) practices has garnered attention for its impacts on nutritional health. For Indigenous people in multiple contexts, the displacement of TFs is associated variously with underweight, stunting, micronutrient deficiencies, overweight, diet-related chronic diseases and the intergenerational effects of malnutrition, especially when coupled with poverty [5, 8, 13, 14]. Researchers observe a disproportionately large prevalence of simultaneous nutrient inadequacies and excesses, dubbed the double burden of malnutrition, among Indigenous people in Canada, Brazil and Guatemala [15–18]. This trend is also clear among Ecuador’s Indigenous people, who have the nation’s highest prevalence of micronutrient deficiencies and are also experiencing increasing prevalence of overweight and obesity [19]. Further, declines in TF production practices may lead to ecological degradation that not only sets off a feedback cycle of further decline in TF practices, but can also trap farmers in poverty [20] and perpetuate food insecurity [21]. In light of such evidence, supporting diverse TF practices is emerging as an international prerogative [5, 7, 14].

Identifying the factors that may actively promote TF practices begins with understanding how TFs are obtained, and who is producing or consuming them. Some TFs are available for local consumption through conventional market purchase [22]. For the many TFs that markets neglect, own production, wild harvest and hunting, and the social economy (local trade, including direct purchase, barter and gifting) are primary forms of access, and the people that continue to obtain food from these subsistence practices are better positioned to consume TFs [5, 23–25]. Following suit, the most widely recognized stewards of TF practices are Indigenous people [4, 5, 26], older generations [4, 25–27], and the rural poor [26, 28]. Similarly, living in remote areas is associated with stronger TF practices, and especially wild harvest, due to reduced opportunities for market integration or marginal ecological conditions that necessitate better-adapted crops [26, 28, 29]. High inter- and intraspecies diversity is also integral to most traditional agricultural strategies [1, 30]. These correlates help to understand where and among whom we might expect to observe TF practices, but they do not necessarily offer reasonable courses of action. For example, it makes no sense to suggest that people be isolated, old and poor in the name of supporting TF practices.

In the Ecuadorian context, a possible proactive driver of TF practices is the growing movement toward agroecological farming. Agroecology applies ecosystem science to agriculture and uses biodiversity, symbiotic relationships, biological controls, and a healthy soil microbiome to support productive and environmentally regenerative farming [30–32]. A growing number of marginalized, resource-poor and Indigenous farmers in Ecuador and around the world have adopted agroecology because of its compatibility with traditional agricultural systems [30, 33, 34].

While agroecology in Ecuador emerged largely out of a need for more environmentally sustainable agricultural practices [30] and as a means to prevent pesticide poisoning [35, 36], the Indigenous resistance movement further saw agroecology as an opportunity to maintain cultural sovereignty in a number of spheres, including agriculture and food [33, 36]. While agroecology in Ecuador eventually spread to include farmers of non-Indigenous identity, today’s “agroecological” identity is largely entangled with Indigenous traditions and objectives. Because agroecological farming has much in common with traditional farming strategies, the distinguishing characteristic of agroecological farmers is typically their membership in an association that participates in an alternative food network such as a farmers’ market [33, 37]. The agroecology movement’s close connections with Indigenous identity and its embrace of TF practices make it a unique space of inquiry for measurable impacts on TFs. Agroecology’s potential to promote TFs is particularly relevant given its ongoing expansion as the predominant framework for connection among food-oriented social movements and peasant farmer organizations across the world [30, 34].

In this study, we aim to understand the factors that are associated with and may serve to promote TF agricultural and dietary practices among farmers in the Ecuadorian highlands. We assess the diversity of production and consumption of several indicator traditional foods, as well as their frequency of consumption. Further, we assess consumption of wild harvested foods. Finally, we explore the relationship between agroecology and TF practices by comparing TF practices among farmers that do and do not participate in the agroecology movement.

## Methods

### Study site and population

This study was conducted in the Imbabura province of Ecuador’s highland region, where people live and farm in areas ranging from around 500 to 3500 m above sea level. The rapidly-changing ecosystems associated with this drastic topography are favorable for diversified production across climatic niches, but also lead to soil erosion and infrastructure challenges on steep slopes [38]. As such, the steepest, most remote, and otherwise most marginal lands are home to the highest poverty rates, with some rural communities in the province reaching 99.8% prevalence of poverty by basic needs [39]. Farmers in these communities are predominantly smallholders, with many managing less than 1 ha of land. Imbabura is nationally distinguished as a cultural hub for Kichwa Indigenous people, and 25.8% of the population identifies as Indigenous [40]. Of Imbabura’s Indigenous people, 86.6% live in rural areas [41], where they utilize agriculture for both own-consumption and sale, as well as partake in other livelihood strategies.

The study population exclusively comprises female smallholder farmers, as women are primarily responsible for food preparation, and it includes women from all six of Imbabura’s cantonal districts. Farmers were selected from two categories: (i) agroecological farmers: farmers who participate in agroecological market associations and are selected at random from a list of association participants, which was generated with local partners prior to recruitment; and, (ii) reference farmers: farmers who are randomly-selected neighbors of agroecological farmers and do not participate in agroecological market associations. One reference farmer was sampled for every second agroecological farmer. The sample size is larger for agroecological farmers to address additional study objectives that are beyond the scope of this article. Interventions promoting agroecology in the region primarily targeted marginalized, Indigenous communities [36], meaning that both the agroecological farmers and their reference neighbors in the present study tend to be from such communities. Farmers from the study population are semi-commercial, meaning that they produce food for own consumption but also aim to generate a surplus for sale.^2^

### Study instruments

**Fig. 1:**
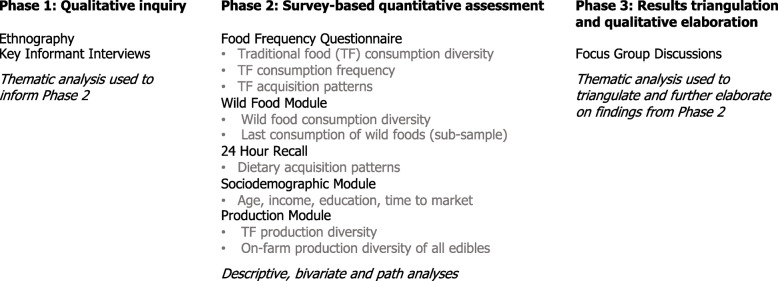
Overview of study phases, instruments, variables and analyses

We employed a three-phase exploratory and sequential mixed methods approach [43], summarized in Fig. 1. The first phase employed ethnography and key informant interviews [36]. This informed the design of the second phase, which was a cross-sectional survey conducted in Imbabura province from July 2017–October 2017 with 91 female farmers (61 agroecological and 30 reference farmers). The survey included a food frequency questionnaire on the consumption and acquisition of indicator TF products, with specific modules on wild food consumption, production diversity of edible foods and livestock, and sociodemographic characteristics. Further, it included a quantitative, multi-pass 24-h dietary recall [44] that gathered information on the source of each food item. The survey was developed to accommodate multiple study objectives and included additional modules that are not addressed here. The survey materials used in this study are provided in Additional file 1. Surveys were conducted in farmers’ homes in Spanish. For farmers who spoke only the local Indigenous language, Kichwa, a family member was recruited to translate. Finally, the third phase deployed eight focus group discussions to triangulate results, as further detailed in the section “results triangulation.”

### Traditional food practice variables

We follow the consumption and production of products that are socio-culturally and bio-culturally considered to be traditional in our study context [1] in order to explore TF practices. Specifically, we assess: (i) TF consumption diversity, (ii) TF consumption frequency, (iii) TF production diversity, and (iv) wild food consumption diversity.

TF consumption diversity and TF consumption frequency are measured from the survey’s food frequency questionnaire (FFQ). The FFQ contains 12 indicator foods^3^ selected following consultation with local experts to include both TF products that are commonly consumed and easily accessible in markets (Andean lupine, *melloco*, quinoa, sweet potato, *zanahoria blanca*) as well as those that are locally recognized as underutilized (amaranth, yacón, *oca*, *mashua*, amaranth leaf, quinoa leaf). We also include *chulpi*, which is an increasingly underutilized maize cultivar [45]. The selected indicators were chosen to also represent the multiple climatic niches in Imbabura province. The sum of indicator TFs consumed produces the TF consumption diversity variable, with a maximum value of 12. Because many of the indicator TFs are only available during specific seasons, we used the frequency of consumption over the reported period of availability (in months) to calculate the annual frequency of each TF. We then summed frequencies of all TFs to obtain the aggregate annual frequency of TF consumption, or TF consumption frequency.

TF production diversity is a count of the different indicator TFs produced on the farm in the past year, with a maximum of 11 products. This is fewer than the maximum for TF consumption diversity because quinoa seed and quinoa leaf are both from the same plant; however, because amaranth seed and amaranth leaf are obtained from distinct varieties, these are maintained separate.

We calculate wild food consumption diversity based on the wild foods that farmers report consuming in an open recall with no specific timeframe. We only consider caloric wild edibles, meaning we ignore plants used exclusively as herbs or teas. For a subset of farmers (*n* = 22), we also queried for the moment of most recent consumption for each product consumed.

### Sources of TFs and general dietary acquisition patterns

To understand how participants obtain each TF, the FFQ also queried for the most common source of acquisition. Similarly, to understand food acquisition practices more generally, we use the item source data from 24-h recalls to calculate the caloric share of the diet (as a percentage of total calories) that comes from distinct food sources. For both TF acquisition and overall dietary acquisition, reported sources were grouped into three categories: harvest (own-production or wild harvest); social economy (barter, gifting, or direct purchase from other farmers); and, conventional market purchase (wet markets, supermarkets, grocers, corner-stores, other).

### Sociodemographic and agricultural variables

We assess age, income, time to market, on-farm production diversity and food acquisition practices as potential correlates of TF practices. Age, monthly income (USD), time to market and education completed are participants’ self-reported values. Household size is used to calculate monthly income per capita. We calculate farm production diversity as a list-based species richness count of caloric edible products (excluding spices and herbs) as well as livestock.

### Statistical analysis

We performed bivariate analyses to compare agroecological farmers and their reference neighbors. We use Pearson’s and Spearman’s correlations (for parametric and non-parametric variables, respectively) to explore relationships between TF production diversity, TF consumption diversity, TF consumption frequency, and wild food consumption diversity, as well as their relationships with other potential correlates. Farming category is input as a dummy variable (reference = 0, agroecological = 1) and the ordinal variable on education completed is treated as continuous (none = 0, partial primary = 1, complete primary = 2, partial secondary = 3, complete secondary = 4, post-secondary = 5). Because this study explores human dietary and production behavior, we defer to behavioral statistics to characterize effect size, with R-values near or above 0.5 (R^2^ = 0.25) considered as a large effect size and R-values near or above 0.3 (R^2^ = 0.09) considered a medium effect size [46]. We then input the strongest correlates into a path analysis to better understand predictors of TF practices. We did not include wild food consumption diversity in path analysis because we did not identify likely correlates for inclusion in the model. Given our sample size, we assessed goodness of fit using the standardized root mean squared residual, with values below 0.08 considered adequate, as well as the root mean squared error of approximation, with values below 0.06 considered adequate [47]. As often occurs in behavioral research, one of our path analysis dependent variables, TF consumption frequency, is not normally distributed. Although path analysis is intended to function with normally-distributed variables, parameter estimates generally remain valid even with non-normal data; however, non-normal data may produce biased standard errors [48]. Further, 24-h recall data was missing for one farmer, producing an agroecological sample size of 60 for some variables. All analysis was conducted using SAS software, version 9.4.

### Results triangulation and qualitative elaboration

We implemented focus group discussions (FGDs) [43] to assess whether farmers’ perceptions converged with quantitative results and to explore how farmers explain the drivers behind the results. Further, these served to return study results to local communities. In March and April 2019, we conducted eight FGDs with 128 total participants. Participants were from the eight agroecological associations whose members had participated in the quantitative study. FGDs were carried out in Spanish, or in Spanish with Kichwa translation by the association leader on an as-needed basis. Farmers voted on “what type of farmer consumes more traditional foods,” with possible answer choices of: agroecological, reference, or both consume equally/uncertain. They were then asked to explain their decision. Then, survey results regarding TF practices were revealed and compared to results from the voting activity. Farmers were asked if they agreed with the findings, and time was allotted for open discussion. Voting activity answers were tabulated, and notes on all other discussion were taken by hand. FGDs were not conducted with reference farmers because reference farmers are not necessarily aware of agroecology and do not self-identify as counterfactuals to agroecological farmers, making it inappropriate to elicit comparisons between the two groups.

## Results

### Traditional food practices among agroecological and reference farmers

Table 1 describes the sample and compares agroecological and reference farmers on study variables. Agroecological farmers have greater TF production diversity, TF consumption diversity and TF consumption frequency than their reference neighbors. The two groups perform equally on wild food consumption diversity. We detected compelling differences in production diversity and food acquisition practices, but not in sociodemographic characteristics.

**Table 1:** Sample description and comparison of agroecological (*n* = 61) and reference (*n* = 30) farmers on study variables

	Descriptive measurements	Comparison by farmer category	
	Pooled sample	Agroecological	Reference
	mean [SD] or percent	mean [SD] or percent	mean [SD] or percent
Traditional food (TF) practices			
TF production diversity (0–11 products)	4.7 [2.5]	5.7 [2.3]***	2.8 [1.9]
TF consumption diversity (0–12 products)	7.5 [2.0]	8.3 [1.7]***	5.9 [1.6]
TF consumption frequency (annual)	221 [182]	260 [193]	144 [129]
*median (interquartile range)*	*164 (82–301)*	209 (130–351)***	*102 (56–180)*
Wild food consumption diversity (products)	7.5 [3.1]	7.7 [3.0]	7.0 [3.2]
Sociodemographics			
Age (years)	45 [13]	46 [13]	42 [13]
Monthly income per capita (USD)	92 [89]	87 [81]	100 [105]
*median (interquartile range)*	*67 (37–110)*	*61 (37–110)*	*85 (40–109)*
Time to market (minutes)	47 [36]	49 [35]	43 [38]
*median (interquartile range)*	*40 (30–60)*	*38 (30–60)*	*43 (20–50)*
Education completed			
None or partial primary	44%	39%	53%
Primary or partial secondary	38%	43%	30%
Secondary or post-secondary	18%	18%	17%
Farm production diversity (products)	39 [16]	45 [15]***	28 [14]
Share of total calories acquired from diverse sources			
Conventional markets (0–100%)	52 [27]	44 [23]***	69 [25]
Harvest (0–100%)	27 [24]	32 [24]***	17 [19]
Social economy (0–100%)	20 [24]	23 [24]	13 [23]
*median (interquartile range)*	*12 (0.2–31)*	17 (6–34)***	0.3 (0.0–16)

For continuous variables, mean is reported with standard deviation. For variables with non-parametric distributions, median and interquartile range are also reported. Frequency is reported for categorical variables. Share of total calories is based on an agroecological sample size of 60, due to missing information. Difference tested between agroecological and reference farmers with Student’s t-test, Mann-Whitney U-test or Chi-Squared test depending on variable distribution and type. *, ** and *** indicate significance at the 10, 5 and 1% levels, respectively.

Supplemental Table 1 (Additional File 2) shows the consumption prevalence, frequency and most common acquisition source for each TF for the pooled population and by farmer group. Agroecological farmers were much more likely to consume underutilized TFs (amaranth, yacón, oca, mashua, amaranth leaf, quinoa leaf) than reference famers, and consumed even the most common TFs (quinoa seed, lupine) at a greater frequency. Among both groups, indicator TFs are most commonly acquired from harvest and most rarely from market purchase. Underutilized TFs are never or very rarely purchased from markets. However, agroecological farmers are more likely than their counterparts to obtain TFs from harvest, and reference farmers are more likely than their counterparts to obtain TFs by means of market purchase. Reliance on social economy for TFs is similar between the two groups

All farmers consume at least one wild food, and on average, they consume between 7 and 8. Wild foods and their consumption prevalence are shown in Supplemental Table 2 (Additional File 2). In the sub-sample of most recent wild food consumption, 32, 23, 27, and 14% did so in the past day, week, month and year, respectively, with only 5% having not consumed a wild food in the past year.

### Correlates and pathways toward traditional food practices

Correlations among TF practices and with other variables are summarized in Table 2. The strongest correlations appear among the four TF practices themselves, as well as with farm production diversity and farmer category. We identified no correlations between TF practices and market distance and only weak, inconsistent relationships with age, income or education. Farmers that obtain a higher share of their food by conventional market purchase tend to have weaker TF practices, whereas those that obtain a higher share of their food from non-market sources (harvest and social economy) tend to have stronger TF practices.

**Table 2:** Correlates of traditional food practices

	TF Production Diversity	TF Consumption Diversity	TF Consumption Frequency	Wild Food Consumption Diversity
Traditional food (TF) practices				
TF consumption diversity	0.61***			
TF consumption frequency	0.33***	0.51***		
Wild food consumption diversity	–	0.30***	–	
Sociodemographics				
Age	0.24**	–	–	–
Monthly income per capita	−0.21**	–	0.28***	–
Time to market	–	–	–	–
Education completed	–	–	0.25*	–
Farmer category (agroecological)	0.54***	0.57***	0.35***	–
Farm Production Diversity	0.58***	0.51***	0.40***	0.24**
Caloric share of diet acquired from diverse sources				
Conventional markets	−0.38***	−0.41***	−0.34***	–
Harvest	0.28***	0.30***	0.28***	–
Social economy	0.20	0.23**	–	–

Correlations are reported using Pearson’s or Spearman’s Rho (R), according to variable distribution. Farmer category is a dummy variable with agroecological set at 1 and reference at 0. Education completed is treated as a continuous variable with values from 0 (none) to 5 (post-secondary). Correlations with R < 0.20 are considered too small to be meaningful and are thus removed for clarity. *, ** and *** indicate significance at the 10, 5 and 1% levels, respectively.

**Fig. 2:**
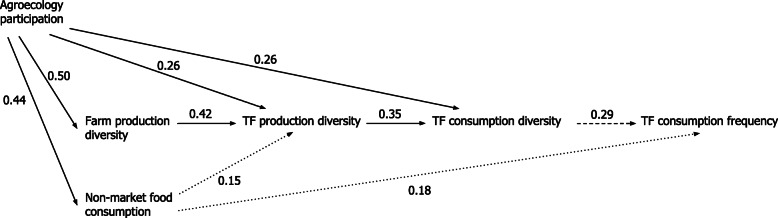
Pathways to traditional food (TF) practices. Standardized estimates for direct effects on traditional food production and consumption are represented with arrows. Dotted lines, dashed lines and solid lines indicate significance levels at 10, 5 and 1% respectively

Figure 2 shows the significant pathways resulting from path analysis, and Table 3 details all path non-standardized and standardized estimates. The modeled pathway shows that higher TF production diversity predicts higher TF consumption diversity, which in turn predicts higher TF consumption frequency. Model estimates suggests that it would take four additional products in TF production diversity to gain an increase of one product to TF consumption diversity. In turn, each additional product in TF consumption diversity predicts 26 additional instances of consumption to the annual TF consumption frequency. Total on-farm production diversity acts on this pathway through TF production diversity, wherein an additional 16 products on the farm predict one additional indicator TF in production. Agroecology participation acts on the pathway through both TF production diversity and TF consumption diversity, contributing an increase of about one product to both TF production and consumption. The share of foods obtained from non-market sources is associated with TF consumption frequency, although the association is not as strong.

**Table 3:** Path analysis estimates for traditional food (TF) production and consumption patterns

Pathway		Path estimate [SE]	Standardized path estimate [SE]	*P*-value
Effects on TF consumption frequency				
TF consumption diversity		26.28 [12.10]	0.29 [0.13]	0.025
TF production diversity		−1.40 [9.66]	−0.02 [0.13]	0.885
Farm production diversity		1.05 [1.36]	0.09 [0.12]	0.439
Agroecology participation		8.68 [48.69]	0.02 [0.13]	0.859
Non-market food consumption		1.22 [0.74]	0.18 [0.11]	0.091
*R-square*	0.1995			
Effects on TF consumption diversity				
TF production diversity		0.27 [0.08]	0.35 [0.10]	0.000
Farm production diversity		0.02 [0.01]	0.15 [0.10]	0.135
Agroecology participation		1.07 [0.41]	0.26 [0.10]	0.008
Non-market food consumption		0.01 [0.01]	0.12 [0.09]	0.171
*R-square*	0.4779			
Effects on TF production diversity				
Farm production diversity		0.06 [0.01]	0.41 [0.09]	0.000
Agroecology participation		1.38 [0.53]	0.26 [0.10]	0.008
Non-market food consumption		0.01 [0.01]	0.15 [0.09]	0.082
*R-square*	0.4349			
Effects on farm production diversity				
Agroecology participation		17.43 [3.20]	0.50 [0.08]	0.000
*R-square*	0.2500			
Effects on non-market food consumption				
Agroecology participation		24.81 [5.39]	0.44 [0.09]	0.000
*R-square*	0.1925			
SRMR	0.0110			
RMSEA	0.0000			

Path estimates and standardized path estimates are shown with standard error (SE) in brackets. SRMR is the standardized root mean square residual. RMSEA is the root mean squared error of approximation.

While correlation and path analyses show a relationship between production and consumption of TFs in general, we find that the extent of this relationship varies from one indicator TF to another (Supplemental Table 3 [Additional File 2]). With the exceptions of quinoa and Andean lupine, farmers that produce a given indicator TF are more likely to consume it and to consume it more often.

### Results triangulation and qualitative elaboration

Table 4 shows that across all focus group discussions (FGDs), participants perceived that agroecological farmers consume more TFs than their reference farming neighbors. FGD participants also perceived survey findings to be accurate.

**Table 4:** Agroecological farmer perceptions on what type of farmer consumes more traditional foods

Focus group number	n respondents	Prevalence of response choice		
		Agroecological farmers consume more	Reference farmers consume more	Both groups consume equally / uncertain
1	19	89%	5%	5%
2	17	59%	12%	29%
3	12	83%	0%	17%
4	17	59%	12%	29%
5	16	81%	13%	6%
6	11	82%	18%	0%
7	15	60%	33%	7%
8	12	75%	17%	8%
Aggregate	119	73%	13%	13%

Responses to focus group discussion (FGD) voting activity on “What type of farmer consumes more traditional foods?” The aggregate prevalence is the prevalence of responses across all FGDs.

Asked to explain why agroecological farmers consume a greater diversity of TF products and with more frequency, all eight FGDs spontaneously produced answers similar to “because we produce more traditional products.” Farmers in six FGDs explained that they produce more TFs in response to consumer demand in the agroecological market. One farmer and market president elaborated:

*“With the* Que Rico Es *[civil society responsible consumption] campaign, one objective is to reposition traditional products. In the [agroecological] markets, the consumer began to understand and request these products, and the farmers also began to assimilate them in their diets. Traditional products are nothing new for the most conscious consumers, and these are the consumers that come to our market.”*

Similarly, some participants credited NGOs and Indigenous federations for their positive influence on TF practices for both farmers and clients involved in agroecological markets. FGD participants identified the role of the agroecological market in strengthening the cultural value that they place on TFs and informing their understanding of TF medicinal or health properties. Many farmers expressed that agroecology strengthened their interest in reclaiming Indigenous identity, and they saw utilizing TFs as a means of doing so. One farmer was met with resounding agreement when she stated, “Since being in the [agroecological] market, we value traditional foods more. Before, we were not like this.”

In further discussion on the importance of TFs, several farmers told stories about how reclaiming TFs allowed them to re-discover the foods of their childhood, and they reminisced on the diverse shapes, colors and flavors of lost varieties. Similarly, one farmer expressed that planting TFs is a means of respecting and reconnecting with his ancestors who developed these products through generations of seed selection. Others saw TFs as a strategic part of agroecological farming, given their pest resistance, low water needs, and adaptability to marginal lands.

Other farmers found TFs to be an important means of supporting nutritional health. Some sustained that TFs contain more vitamins and minerals than “modern” foods, which they saw as the vectors of overweight and disease. Women in particular saw TF preparation as necessary “for the health of the children,” despite requiring more effort to prepare. Discussions tended to emphasize the importance of TFs for children and younger generations, and make reference to healthy growth.

## Discussion

### The state of traditional foods in rural Imbabura diets

Traditional foods remain a part of daily life for farmers in our study population, but there is no bar to gauge how much traditional food consumption is “enough” to curb TF displacement and mitigate the nutrition transition toward foods that contribute to a double burden of over- and undernutrition. Most farmers consume at least half of the indicator TFs assessed, and they consume them often: agroecological farmers report consuming indicator TFs 260 times a year, and reference farmers do so 144 times a year. All farmers continue to practice wild harvest to some extent, and most do so on a weekly basis. TF consumption appears more alive in this farming population than in other spaces in the country; for example, a recent representative study in three Ecuadorian highland cities found that only 19% of participants consumed either quinoa, amaranth or Andean lupine more than three times per month [49]. The comparable figures in our study population would be 60% of reference farmers and 85% of agroecological farmers. Even the indicator TFs that we selected because they are locally recognized as underutilized (amaranth, yacón, oca, mashua, quinoa leaf, amaranth leaf) are all still present to some extent in our study population’s diets. Some of these products are receiving attention for their potential to support dietary health. For example, amaranth seed is recognized for its protein and lipid profiles [50], and amaranth and quinoa leaves are green leafy vegetables with high concentrations of nutrients that are of special concern in the Ecuadorian rural population, notably vitamin A, iron, calcium, zinc and vitamin C [19, 50, 51]. Even though some of these products are only marginally alive in the diets of reference farmers (i.e., with median consumption of only once yearly), they point to opportunities to strengthen the use of endogenous foods to support nutritional health.

### Opportunities for traditional food promotion

Our analysis suggests that TF consumption is associated with TF production. This is no surprise in light of the expanding literature on the pathways between production and consumption, and namely production diversity and dietary diversity [52]. Indeed, we find that farmers that grow a given TF are not only more likely to consume it, but they also consume it more frequently. Some underutilized TFs are exclusively obtained from own harvest. For other TFs, farmers who do not produce them obtain them from farmers who do, relying on social economy transactions such as barter or direct purchase. That these underutilized products are never purchased at markets is likely a consequence of their reduced availability [53], and signals the importance of the social economy in filling supply gaps.

The diversity of TF products grown on the farm is associated with higher overall farm production diversity of edible products. Nevertheless, it is unlikely that increasing agrobiodiversity alone would guarantee an increase in TF production diversity. Instead, the association we detected may reflect adherence to more traditional cropping systems, which depend on relatively high agrobiodiversity [1], or it may be a reflection of the diversity supported by the ecological niche. While there may not be a direct causal relationship between overall farm production diversity and TF production diversity, the two may be mutually reinforced as farmers and organizations aim to increase farm production diversity for ecological, productive and nutritional reasons [32]. Doing so by targeting TF production diversity may be particularly relevant for nutrition-sensitive agriculture initiatives, given that TFs are shown to simultaneously contribute to agricultural resilience, food access [54–57] and to dietary intake of key macronutrients, micronutrients and phytochemicals [56–61], and they further play a protective role against chronic diseases [6, 8, 59].

We further find that farmers whose diets rely less on conventional markets and more on own harvest or the social economy maintain stronger TF practices. Other scholars similarly discuss the importance of non-market subsistence practices such as own production and local trade in conserving traditional crops [5, 23]. In contrast to other studies [4, 26, 28, 29], market distance, income and age did not emerge as strongly or consistently associated with TF practices among our study population. This means that in this context, TF practices are not merely a relic of the most isolated, impoverished and aging—or in short, marginalized—people, as public opinion has long perceived them to be [7]. In the development literature, practices that are the purview of the most marginalized people, and especially of subsistence-oriented farmers, tend to be discussed as “coping” or “adaptive” strategies driven by reactive necessity rather than proactive agency [62]. In contrast, the fact that we detected an association with reliance on non-market food sources but did not detect a strong association with marginalization implies that TF practices in our study population are not merely a reaction to adverse conditions. Possibly, farmers may be participating in a globalized cultural shift toward re-valorization of TFs, as has been described in Europe [63]. Doing so, some may even perceive TF practices as active agents in strengthening cultural identity and food sovereignty [36].

### Agroecology as an incubator for traditional food promotion

Agroecological farmers unambiguously perform better than their reference neighbors on three of the four TF practices assessed. They produce twice as much TF diversity, consume 40% more TF diversity and consume TFs 80% more often compared to their reference counterparts. In our path analysis, participation in agroecology was directly associated with both TF production diversity and TF consumption diversity, leading to a downstream association with TF consumption frequency. While we did not measure changes over time, agroecological farmers emphatically identify their participation in agroecological markets as the drivers of increased TF production and consumption, pointing to agroecology as a means to strengthen TF practices. Moreover, the strongest differences in consumption of specific TFs appear precisely in those that are locally recognized as underutilized. Agroecology may thus be key for reclaiming at-risk TFs in this region and re-inserting them into healthy dietary patterns.

Part of the reason why agroecological farmers in our population perform so much better on TF practices may be because agroecology explicitly promotes farm production diversity and reliance on non-market food sources [34, 36], which are correlates of TF practices. Yet even when these are held constant, agroecology participation still shows an association, suggesting that other forces are at play. Focus group discussions help clarify these unknowns, identifying two additional potential drivers that may motivate agroecological farmers to increase their TF practices.

First, the social environment of the agroecological market association may drive farmers to produce and consume TFs for their nutritional properties, taste, agricultural resilience, cultural value and even aesthetics. While such convictions around TFs are also found among other farmers in Northern Ecuador [64], the social encounters in agroecological spaces appear to further concentrate these convictions by inserting TF practices into social norms that strengthen a shared cultural identity. Further, they seem to embed TF consumption into the moral impetus of feeding healthy food to the family. The importance of these socially-driven elements in guiding TF practices is consistent with dietary behavior models that find food decisions to be informed by “affective” components, including feelings and emotions, moral obligations, and social norms and pressures [65].

Second, focus group discussions also identified the specialized consumer demand for TFs in agroecological markets as a potential driver of TF practices among farmers. Other studies on TFs similarly find that consumer demand-driven value chains influence TF production [66, 67]. However, discussion participants further sustained that when they grow TFs for sale, they also increase their own consumption. These flows of influence are probably bidirectional, given that agroecological farmers’ associations played an important role in the emergence of a nation-wide campaign to form “responsible” consumers that seek out traditional Andean crops as well as nutritious, socially just and ecologically sustainable food [68, 69]. As such, there appears to be a feedback loop between agroecological market farmers and clients in forming affective spaces [36] that support traditional foods.

### Wild harvest

Wild harvest appears to be a common practice among our study population, even though most of the harvested foods are consumed in small amounts as condiments or snacks. As far as we can tell, wild food consumption diversity among our study population is not associated with age, income, distance to markets, food acquisition strategies or agroecology participation. This relative democratization is compelling given that wild foods can be remarkably nutritious, but also remarkably neglected and underutilized [7, 70, 71]. This combination often relegates wild foods to coping strategies for the poorest of the poor and erroneously dismisses them as “famine foods” [7, 70]. While we were unable to detect plausible pathways promoting wild food consumption, we find that people who consume a greater diversity of TFs in general also consume a greater diversity of wild harvested products, potentially signaling similar drivers for these two dietary outcomes. While our findings suggest that wild foods have not been prioritized by the local agroecology movement, its unique affective and commercial spaces may hold the enabling conditions to effectively promote wild foods.

### Internal and external validity of findings

We believe a word of caution is warranted regarding our data on TF consumption frequency, given the cognitive recall difficulties that beleaguer FFQs [72] as well as the added complexity of seasonality [73].^4^ However, farmers participating in focus group discussions corroborated the detected pathways between TF production and consumption. This triangulation between qualitative and quantitative methods gives us more confidence in our findings, despite the relatively small sample assessed in the survey. Nevertheless, we only conducted FGDs with agroecological farmers and we are uncertain of the subjective biases at play. FGDs were also key for identifying farmers’ perceptions of causality between agroecology and TF practices. Moreover, path analysis has the advantage that it can identify likely chains of influence, even with cross-sectional data [74]. While neither the subjective experiences of farmers nor the results of path analysis are sufficient to definitively establish causality, the triangulation of the two strengthens the internal validity of our results. Nevertheless, our study is limited to a single region, and we recognize that many contextual factors could affect external validity. Not only is agroecology a term that embraces many local expressions [30], but other factors that are subject to broad variation include the cultural presence of TFs, ecological context, food acquisition patterns, gender norms around food and many more. Rather than providing a proscriptive formula for strengthening TF practices, it is our hope that we shed light on how these possible paths can play out, recognizing that they will likely be different in other localities.

## Conclusions

In the Ecuadorian highlands, traditional foods (TF) remain a routine part of rural life to a certain extent, but for some TF products, production and consumption decline is a compelling concern. Meanwhile, the nutrition transition away from traditional diets and toward calorie-dense, micronutrient-poor foods marches forward, undermining Indigenous health [2, 75]. Aiming to understand how TF practices may be strengthened, we found evidence supporting a pathway between the production of TFs and their consumption. Key starting points on this pathway appear to be higher farm production diversity of edible products and a stronger reliance on non-market food sources, namely foods from own harvest and from the social economy. Just as interesting as the correlates of TF practices are the non-correlates. Older age, lower income, less education, and greater market distance do not generally predict TF practices in this context. This is cause for optimism, in that it suggests that TF practices are not an exclusive relic of marginalized populations, but rather a dynamic part of the food habits of relatively diverse farming populations.

Agroecological farmers in our study site drastically out-perform their neighbors on TF practices. This may be because agroecology promotes farm production diversity and reliance on non-market foods [34, 36], thus enabling the pathway we identified for TF promotion. However, agroecology also appears to support TF practices in other ways. First, the social spaces surrounding agroecological associations intensify affective (e.g. emotional) relationships with TFs by emphasizing their cultural, health and sensory qualities. Moreover, agroecological markets place farmers in specialized value chains where there is consumer demand for TFs. Importantly, these factors are likely to be locally specific and cannot be copy-pasted to other contexts. Nevertheless, the diversity of ways in which agroecology interacts with TF practices provides hope that it may enhance the role of TFs in the diet without separate investments of capital. It may further be strategic because it is already a rapidly growing global movement [30] with emphatic buy-in among Indigenous people and the rural poor [33, 34, 36], who disproportionately face a double burden of nutrient deficiencies and excesses [2, 5, 21]. Given the growing body of evidence that links traditional diets to the mitigation of the nutrition transition, stronger food security and healthier nutritional status [5, 6, 12, 57–61], we hope the pathway we identified serves to inform effective strategies for TF promotion.

## Supplementary Information

**Additional file 1.** This file contains the survey material used in this study.

**Additional file 2:** This file contains **Supplemental Tables 1**, **2** and **3**.

## Data Availability

The datasets used and/or analyzed during the current study are available from the corresponding author on reasonable request.

## Abbreviations

FGD: Focus group discussion
TF: Traditional food
FFQ: Food frequency questionnaire
SE: Standard error
RMSR: Root mean square residual

## Footnotes

1 We follow Johns and colleagues in using “traditional” as a qualifier for products and practices that are defined both socio-culturally and bio-culturally following a period of historical continuity [1].

2 Pingali and Rosegrant offer a useful discussion on semi-commercial farming [42].

3 Latin names for indicator foods are as follows: Quinoa: *Chenopodium quinoa*; Andean lupine: *Lupinus mutabilis*; Melloco: *Ollucus tuberosus*; Mashua: *Tropaeolum tuberosum*; Oca: *Oxalis tuberosa*; Zanahoria blanca: *Arracacia xanthorrhiza*; Yacón: *Smallanthus sonchifolius*; Chulpi: *Zea mays amylosaccharata*; Sweet potato: *ipomea batata*; Amaranth: *amaranthus spp.* Yacón is known locally as jicama, but we use the regional term yacón in order to avoid confusion with *Pachyrhizus erosus.*

4 Farmers in our study population would often report that they ate a given product “every day while it’s available”, which, for a product that is available for two months, would lead to an unrealistic estimated frequency of 60 during that time period. While we believe this inflation would be equally distributed across both agroecological and reference farmers, we do not have a reliable means of correction in order to obtain a more accurate TF consumption frequency.
