# Supplementary material for: Promoting traditional foods for human and environmental health: lessons from agroecology and Indigenous communities in Ecuador
Source: BMC Nutr. 2021 Jan 7;7:1. doi: 10.1186/s40795-020-00395-y (PMC7792355; doi:10.1186/s40795-020-00395-y)
Supplement: Supplementary file 1 — Additional file 1. This file contains the survey material used in this study. [file 40795_2020_395_MOESM1_ESM.pdf]

**Additional File 1**

**Introduction to additional file:** This document includes the English translation of the survey questions and instrument protocol utilized for the analyses presented in this article. Protocol notes are denoted in blue italic text. Spanish or Kichwa local names for foods are in italics.

ID Number (NOID)__________ *Codes denote agroecological and reference sample.*

***Interviewer instructions:*** *read the consent script*.

| | |
|---|---|
| Consent: Do you consent to participate in this survey? | ☐ Yes ☐ No (end interview) |
| Enter date | |
| Enter time | |
| First and Last Name | |
| Community | |
| Parish | |
| Canton | |
| Agroecological market | |

| | |
|---|---|
| Latitude | |
| Longitude | |

**CONSUMPTION**

**24 Hour Recall**

First, I would like to ask you about all of the foods or drinks that you consumed yesterday, from the moment that you woke up to the moment you went to sleep.

What was the first thing that you put in your mouth? What was the next...?

***Interviewer instructions:*** *Continue the line of questioning until you get to the point of going to sleep. Afterward, revise by each meal (breakfast, lunch, dinner), and anything consumed between meals. Revise drinks, snacks, desserts, foods eaten on-the-go. Leave space between complex foods to later add more details. Then, ask about the main ingredients in complex foods. After generating the list, continue with the next questions to complete the table.*

Now, for each item you consumed, I would like to know where you obtained it, or the ingredients for it. That is, I want to know if it came from your own harvest, if someone gave it to you, if you traded for it, or if you purchased it. If you purchased it, I would like to know from what commercial location, and in what village or city.

**DIET. 24 hour recall.** *Use response codes to facilitate data entry*

| Moment of the day | Dish | Portion quantity | Ingredients [type, preparation, processing, brand] | Ingredient quantity | Origen |
|---|---|---|---|---|---|
| | | | | | |

Was there anything special yesterday that may have affected your diet? (e.g. party, illness) ____________

**Traditional products**. Now I would like to ask you about specific foods that you may eat, as well as how often you eat them, and where you obtain them.

| | n / day | n / week | n / month | n / year | very rarely | never | unfamiliar with product | duration of availability season | most common origin of product |
|---|---|---|---|---|---|---|---|---|---|
| Quinoa leaf | | | | | | | | | |
| Quinoa (seed/grain) | | | | | | | | | |
| Amaranth leaf | | | | | | | | | |
| Amaranth (seed/grain) | | | | | | | | | |
| Lupine | | | | | | | | | |
| *Melloco* | | | | | | | | | |
| *Mashua* | | | | | | | | | |
| *Oca* | | | | | | | | | |
| *Zanahoria blanca* | | | | | | | | | |
| Yacón | | | | | | | | | |
| *Chulpi* | | | | | | | | | |
| Sweet potato | | | | | | | | | |

In the past year, have you consumed any of the following wild products?.....Are there any that you consumed that are not listed?

| | | | | | | | |
|---|---|---|---|---|---|---|---|
| | *bledo* | | *Lengua de vaca (wagrahayu)* | | *Uvilla de lobo* | | Others: |
| | *nabo de monte* | | *Quinua de monte (allpa quinua)* | | *Taxo de monte* | | |
| | *Rábano de monte* | | *Mora de monte (mora silvestre)* | | *Chimbalo* | | |

| | | | | | | | |
|---|---|---|---|---|---|---|---|
| | *Berro de monte* | | *Uvilla de monte* | | *Mortiño* | | |

**PRODUCTION**

Approximately how much land do you use for farming and livestock? •<1 ha. •1-3 ha. •3-5 ha. •5-10 ha. •>10 ha.

Do you have irrigation? •Yes •No

Of the following animals, which do you have, even if in small quantities?

| | | |
|---|---|---|
| Dairy cows | Alpacas | Quails |
| Cattle for meat | Laying hens | Tilapia |
| Pigs | Chickens (for meat) | Trout |
| Sheep | Ducks | Guinea pig |
| Goats | Geese | Rabbits |
| Llamas | Pigeons | Others: |

Of the following products, which are you currently growing or have you grown in the past year, even if only in small quantities?

| | | | | | | | | | | | | | |
|---|---|---|---|---|---|---|---|---|---|---|---|---|---|
| | Chard | | Onion, *perla* | | Strawberry | | *Castillo* blackbery | | Romanesco | | Watercress | | Guanábana |
| | Slipper gourd | | Onion, *paiteña* | | *Granadilla* passionfruit | | Other blackberry | | Arugula | | *Nabo* | | Arazá |
| | Avocado | | Leek | | *Guaba* | | Andean blueberry | | Rhubarb | | Kale | | *Achotillo* |
| | Hot pepper | | Chive | | Guava | | Orange | | Aloe vera | | Aromatic herbs or plants | | Turmeric |
| | Garlic | | Cherry plum | | Fava bean | | *Naranjilla* | | Agave | | | | |
| | Basil | | Chayote | | Fig | | Loquat | | *Sambo* squash | | ***From warmer climates:*** | | |
| | Artichoke | | Chia | | Fennel | | *Oca* | | Banana passionfruit | | Yuca | | |
| | Alfalfa | | Chirimoya | | Yacón | | Oregano | | Tamarillo | | Cacao | | |
| | Amaranth | | Andean lupine | | Iceberg lettuce | | *Paico* | | Tomato | | Coffee | | |
| | Amaranth | | Cilantro | | Romaine lettuce | | Potato | | Grapefruit | | Mango | | |

| | | | | | | | | | | | | | |
|---|---|---|---|---|---|---|---|---|---|---|---|---|---|
| | Celery | | Green cabbage | | Other lettuce | | Taro | | Wheat | | Mangostine | | |
| | Pea | | Red cabbage | | Lemon | | Turnip | | Cactus fruit | | Peanut | | |
| | Oat | | Brussel sprout | | Lime | | Cucumber | | Grape | | Pineapple | | |
| | *Babaco* highland papaya | | Cauliflower | | Flax | | Pepino | | Groundcherry | | Plantain | | |
| | *Chamburo* highland papaya | | Turmeric | | Lovage | | Pear | | Green bean | | Soy | | |
| | *Chihualcán* highland papaya | | Peach | | Maize | | Parsley | | Carrot | | Tamarind | | |
| | Broccoli | | Asparragus | | Mandarine | | Sweet pepper | | *Zanahoria blanca* | | Banana | | |
| | Sweet potato | | Spinach | | Apple | | Quinoa | | Pumpkin | | *Orito* banana | | |
| | *Capulí* | | Stevia | | Maracuya passionfruit | | Radish | | *Zapote* | | *Caimito* | | |
| | Barley | | Raspberry | | *Mashua* | | Plum | | Zucchini | | Sugar cane | | |
| | Onion, *larga* | | Bean | | *Melloco* | | Beet | | Sorrel | | Ginger | | |

**SOCIOECONOMIC AND DEMOGRAPHIC**

How many individuals live in the household including yourself? ______________

How old are you? ______

| | |
|---|---|
| What level of education have you completed? | Daycare or none<br>Partial primary school<br>Primary school<br>Partial secondary school<br>Secondary school<br>University or post-secondary school |

Does anyone in your household receive the Human Development Bonus? •No •Yes. How many people receive it?___

Adding up the incomes of all the people in the household, what is more or less the total monthly income of the home, without including the Human Development Bonus?
