# Supplementary material for: Promoting traditional foods for human and environmental health: lessons from agroecology and Indigenous communities in Ecuador
Source: BMC Nutr. 2021 Jan 7;7:1. doi: 10.1186/s40795-020-00395-y (PMC7792355; doi:10.1186/s40795-020-00395-y)
Supplement: Supplementary file 2 — Additional file 2: This file contains Supplemental Tables 1, 2 and 3. [file 40795_2020_395_MOESM2_ESM.pdf]

Additional File 2:

Supplemental Tables 1 – 3

Supplemental Table 1: Consumption prevalence and frequency of indicator traditional foods (TF) and their most common sources of acquisition, by agroecological (AE) and reference (R) farmer categories

	TF consumption						Prevalences of TF acquisition sources reported as primary source (%)								
	Prevalence (%)			Annual frequency			Conventional markets			Harvest			Social economy		
	Pooled	AE	R	Pooled	AE	R	Pooled	AE	R	Pooled	AE	R	Pooled	AE	R
Quinoa leaf	22	32***	3	2 [5]	2 [8]	1 [0]	0	0	0	93	96	75	7	4	25
Quinoa seed	96	95	97	24 [40]	36 [40]***	12 [33]	17	10**	31	48	56**	31	35	34	38
Amaranth leaf	20	25*	10	6 [23]	12 [51]	1 [5]	0	0	0	100	100	100	0	0	0
Amaranth seed	19	25**	7	8 [10]	8 [24]	7 [10]	3	0*	14	47	48	43	50	52	43
Andean lupine	99	100	97	52 [28]	52 [28]**	30 [40]	38	23***	69	29	35*	17	33	42***	14
Melloco	94	98**	87	24 [40]	52 [40]*	24 [40]	28	17***	50	30	36	20	42	47	30
Mashua	38	50***	13	3 [11]	3 [11]	1 [26]	16	8***	42	48	55*	25	36	37	33
Oca	84	90**	73	5 [13]	5 [16]	4 [11]	12	9	22	37	36	39	51	55	39
Zanahoria blanca	79	87**	63	4 [11]	4 [11]	3 [13]	6	4	13	61	71***	38	33	25**	50
Yacon	53	63***	33	2 [11]	3 [21]***	1 [1]	0	0	0	66	69	54	34	31	46
Chulpi	63	77***	37	12 [24]	12 [50]	12 [11]	25	17***	47	52	62***	26	23	21	26
Sweet potato	83	88*	73	4 [23]	4 [17]	6 [32]	12	7**	24	42	48*	28	46	45	48
n farmers		60	30			Aggregate prevalence (%)	16	10***	33	48	55***	32	36	36	35

TF consumption prevalence shows the percentage of farmers that report consuming each TF item over the past year. TF consumption annual frequency reports the median frequency of consumption among farmers that consumed the TF, with interquartile range in brackets. The prevalence of certain products among reference farmers, especially quinoa leaf, amaranth leaf, amaranth seed and mashua, may be too low to accurately detect a difference in annual consumption frequency between the two groups. For each TF item, farmers reported their primary source for acquiring the item, and these were categorized into three groups: conventional markets, harvest (including harvest from own production and wild harvest), and social economy (including barter, gifting or direct purchase from another farmer). The aggregate prevalence of each acquisition source is the percentage of times that the source is mentioned as the primary source across all TF items. Differences in prevalence are tested between agroecological and reference farmers with the Chi-square test, and differences in frequency are tested with the Mann-Whitney U-test. *, ** and *** indicate significance at the 10%, 5% and 1% levels, respectively.

Supplemental Table 2: Consumption of wild foods among study participants

Local name of wild food	Alternative local names	Consumption prevalence
Berro		92%
Mora de monte	Mora de árbol	80%
Bledo	Lido, Ledo	71%
Nabo de monte	Nabo de chagra, Chagrayuyu, Nabo de maíz, Alli yuyu, Nabo nacional	69%
Chimbalo		65%
Mortiño		60%
Uvilla de monte		56%
Taxo de monte	Anga taxo	52%
Rábano de monte	Rábano yuyu	48%
Lengua de vaca	Wagrahayu	30%
Uvilla de lobo	Uvilla sin cáscara, Lluchu uvilla, Uvilla macho, Uvilla eljersita	20%
Quinua de monte	Allpa quinua, Panrra	16%
Motilón		14%
Walicón	Chaupalón, Capulí de monte, Chupalulu, Chupalulun, Pinol	11%
Tuna		7%
Ságalan	Ságala	7%
Tocte		7%
Chugonda	Chugunda	4%
Chihualcán	Chamburo silvestre	3%
Sacha piña	Piñuela	3%
Frutilla de monte		2%
Frambuesa silvestre		1%
Machia		1%
Waka mollo		1%
Nopal		1%
Kanayuyo		1%
Manzanita	Niwas	1%

Serote	1%
Uchu moras	1%
Juña foro	1%
Berro de laguna	1%
Verdolaga	1%
Mandarina de monte	1%
Hongos blancos	1%

Table summarizes prevalence of each wild food across all study participants (n=91), excluding wild products identified as used only for brewing as herbal tea or medicinal remedies. Local names are used, with alternative local names identified as equivalent products.

Supplemental Table 3: Relationship between traditional food (TF) production and consumption, by TF Product

TF Item	TF item in production	Prevalence of TF consumption (%)	Odds ratio of TF consumption	Median TF consumption frequency
Quinoa leaf	Yes	35***	7.1	2*
	No	7		1
Quinoa seed	Yes	98	3.6	30
	No	93		24
Amaranth leaf	Yes	39***	10.7	12**
	No	6		1
Amaranth seed	Yes	48***	9.3	12
	No	9		2.5
Andean lupine	Yes	100	not computed	52
	No	98		52
Melloco	Yes	100**	not computed	43
	No	90		24
Mashua	Yes	83***	16.5	6
	No	22		1
Oca	Yes	94*	3.7	17***
	No	80		3
Zanahoria Blanca	Yes	91***	6.7	6**
	No	60		2
Yacon	Yes	74***	6.7	3***

	No	30		1
Chulpi	Yes	87***	6.5	24***
	No	51		5
Sweet potato	Yes	96***	9.8	5
	No	70		3

To compare farmers that do and do not produce a given TF item, differences in prevalence of TF item consumption are tested with the Chi-square test and differences in frequency are tested with the Mann-Whitney U-test. *, ** and *** indicate significance at the 10%, 5% and 1% levels, respectively. Odds ratios indicated as "not computed" occur when a category contains a 0-value. In both cases, this occurs when there is no individual that produces a given TF but does not consume it.
